# Connective Tissue Growth Factor: Regulation, Diseases, and Drug Discovery

**DOI:** 10.3390/ijms25094692

**Published:** 2024-04-25

**Authors:** Meishen Ren, Shanshan Yao, Tienan Chen, Hang Luo, Xiaohui Tao, Hewen Jiang, Xin Yang, Huarui Zhang, Sifan Yu, Yin Wang, Aiping Lu, Ge Zhang

**Affiliations:** 1 Key Laboratory of Animal Diseases and Human Health of Sichuan Province, College of Veterinary Medicine, Sichuan Agricultural University, Chengdu 611130, China; 2 Law Sau Fai Institute for Advancing Translational Medicine in Bone and Joint Diseases (TMBJ), School of Chinese Medicine, Hong Kong Baptist University, Hong Kong SAR, China; 3 School of Chinese Medicine, Faculty of Medicine, The Chinese University of Hong Kong, Shatin, Hong Kong SAR, China

**Keywords:** CTGF/CCN2, regulation mechanism, therapeutic target, drug discovery, CTGF-related diseases

## Abstract

In drug discovery, selecting targeted molecules is crucial as the target could directly affect drug efficacy and the treatment outcomes. As a member of the CCN family, CTGF (also known as CCN2) is an essential regulator in the progression of various diseases, including fibrosis, cancer, neurological disorders, and eye diseases. Understanding the regulatory mechanisms of CTGF in different diseases may contribute to the discovery of novel drug candidates. Summarizing the CTGF-targeting and -inhibitory drugs is also beneficial for the analysis of the efficacy, applications, and limitations of these drugs in different disease models. Therefore, we reviewed the CTGF structure, the regulatory mechanisms in various diseases, and drug development in order to provide more references for future drug discovery.

## 1. Introduction

Connective tissue growth factor (CTGF) is a multifunctional protein, also known as cellular communication network factor 2 (CCN2). It belongs to the CCN family of growth factors [1]. CTGF maintains regular expression levels in normal tissues. Its expression varies in related disease conditions, such as fibrosis, cancers, and atherosclerosis [2]. As a conserved protein, CTGF is a versatile potential therapeutic target that exhibits different functionalities in the growth, development, and maintenance of tissues in the body. CTGF can induce the expression of communication-related genes, such as phosphatidylinositol-3 kinase (PI3K)/protein kinase B (Akt), to promote cell communication [3]. It can also interact with integrins and fibroblast growth factor receptors, such as tyrosine kinases and ERK, leading to signaling activation and the promotion of cell differentiation [4]. Cell proliferation can also be promoted by CTGF in diseases such as fibrosis and cancer. In the hepatocellular carcinoma model, elevated CTGF expression stimulates cell proliferation through the activation of sphingosine-1-phosphate 2 (S1P2)-mediated Yes-associated protein (YAP) signaling [5]. Understanding the functions and regulatory processes of CTGF in different disease models will provide a reference for the development of new drugs in the future.

Growth factors belonging to the CCN family consist of a cysteine-rich matrix protein with an N-terminal secretory peptide and four multifunctional structural domains [6]. These molecules has a complex network of interacting ligands that may affect related signaling pathways [7]. The gene sequence and protein composition of CTGF have been extensively studied, although no full-length crystal structure information has been reported. At the N-terminal, CTGF consists of a signal peptide followed by four major structural domains, including insulin-like growth factor binding protein (IGFBP), von Willebrand factor type C repeat (VWC), thrombospondin type-1 repeat (TSP-1 or TSR), and cysteine knot-containing domain (CT) [8]. The N-terminal of CTGF is composed of the IGFBP and VWC domains, while the C-terminal contains the TSP-1 and CT domains. The N-terminal and C-terminal are separated by a long stretch, namely the Hinge region [9,10]. Researching the interactions between these four CTGF domains and their binding ligands increases the understanding of the effects of CTGF on cellular physiological processes.

In the development of therapeutic drugs for different diseases, many drugs targeting and inhibiting CTGF have been developed, including small-molecule compounds, antibodies, peptides, nucleic acid drugs, and gene therapy drugs [1,2]. We utilized the AlphaFold, DrugBank, and ClinicalTrials.gov databases to obtain information on protein structure, drug chemical structures, and clinical trials. Using the Web of Science Core Collection and PubMed database, a comprehensive search of the literature published between 1997 and 2024 was conducted. This review introduces the structure of CTGF and emphasizes the importance of CTGF as a therapeutic target in various diseases and drug development.

## 2. Interaction to Molecules and Related Signaling Pathways of CTGF Domains

### 2.1. IGFBP Domain

**Figure 1:**
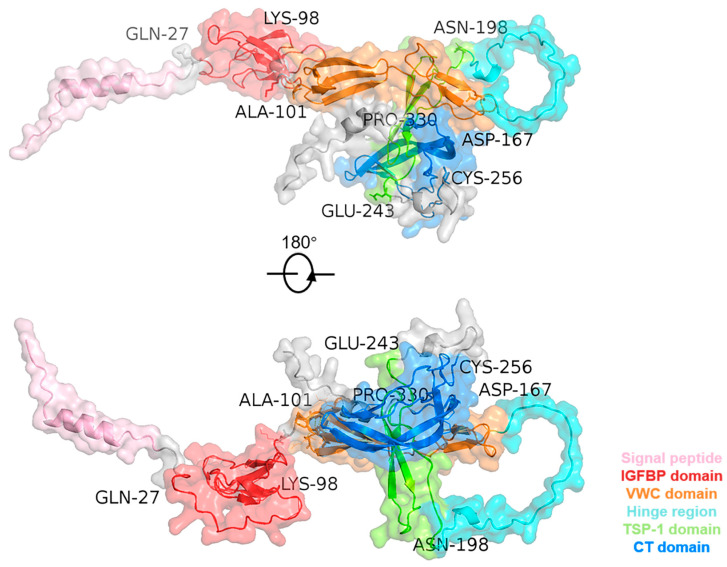
Three-dimensional structure of the CTGF protein. The human CTGF structural PDB file was obtained from the AlphaFold Protein Structure Database (https://alphafold.com/, accessed on 7 April 2024).

CTGF contains four structural domains that differentially regulate mechanisms in disease processes (Figure 1). The four structural domains (IGFBP, VWC, TSP-1, and CT) can interact with various receptors and ligands separately or in combination to affect the activation of their downstream signaling. The IGFBP (domain I, exon 2) is located in the amino acid fragment (Gln27-Lys98) in CTGF with a representative conserved motif GCGCCXXC. The IGFPB domain participates in the effects of several ligands and their downstream pathways. For instance, insulin-like growth factor (IGF) is an essential molecule in cell growth, differentiation, and apoptosis [11]. Due to the similarity of its structure to that of the IGFBP family, the IGFBP domain of CTGF was found to conduct regulation in a similar manner to IGFBP-3 in pathways such as transforming growth factor-β (TGF-β)/Smad, heparin/glycosaminoglycans, and preadipocyte differentiation [12,13,14]. Like IGFBP-7, CTGF binds to IGF with relatively low affinity. This characterization of CTGF causes it to be regarded as a member (IGFBP-8) of the IGFBP family [15]. It was also found to affect the differentiation of osteoblasts and to enhance bone formation [16,17]. As for the other targeting ligands, CTGF binds to aggrecan through the IGFBP and VWC domains to promote the expression level of aggrecan in chondrocytes [18]. The Rab GTPase family protein Rab14 was also found to interact with the CTGF IGFBP domain to induce proteoglycan secretion in chondrocyte cells [19]. 

### 2.2. VWC Domain

The VWC domain at the N-terminal sequence of Ala101-Asp167, which contains the conserved sequences CXXCXC and CCXXC, participates in the regulation of transforming growth factor-β (TGF-β) and the related development of the bone morphogenetic proteins (BMPs) [12,20]. The VWC domain activates several signaling pathways, including the TGF-β, c-Jun N-terminal kinase (JNK), and phosphatidylinositol-3 kinase (PI3K)/protein kinase B (Akt) pathways, which play critical roles in cellular proliferation and differentiation [21]. CTGF directly binds to TGF-β and BMP 2/4 in the extracellular space through the VWC domain. However, the regulatory mechanisms are opposite when the VWC domain binds to TGF-β and BMP separately. CTGF inhibits BMP but activates TGF-β1 signaling. The weak interaction between the CTGF VWC domain and TGF-β1 was not found to antagonize the binding of TGF-β1 to its receptor; rather, it potentiated TGF-β1 receptor binding and signaling [22].

The TGF-β signaling pathway regulates several aspects of cell behavior, including cell proliferation, differentiation, and extracellular matrix production, and plays a crucial role in tissue repair and wound healing [23]. The VWC domain promotes TGF-β signaling by binding to TGF-β receptor II (TβRII) and activating the downstream activation of Smad2/3 signaling. This results in increased collagen synthesis and extracellular matrix remodeling in fibroblasts. In contrast, CTGF is regarded as an antagonist of BMP due to its direct binding through the VWC domain. For instance, CTGF was reported to negatively regulate the activities of BMP-2 and BMP-7 [24,25]. The persistent elevation of CTGF causes a decline in osteoblast maturation and mineralization, regardless of whether the cells are stimulated with BMP-2 [25]. CTGF functions as a negative regulator of BMP-2-induced signaling and the differentiation of osteoblasts. Regarding BMP-7, CTGF upregulation interferes with BMP-7 signal transduction in diabetic kidneys, causing changes in gene transcription, decreased MMP activity, thickening of the glomerular basement membrane, and an increase in albuminuria [24].

In other cases, CTGF binds to aggrecan through its VWC domain, which is related to the production and secretion of aggrecan by chondrocytes [26]. In the conserved family homologs CCN1, the interaction of the VWC domain with integrin αVβ3 is necessary to stimulate matrix metalloproteinase 1 (MMP-1) expression with the cooperation of its IGFBP and TSP-1 domains, which may be the cause of collagen fibril fragmentation and skin aging [27].

### 2.3. TSP-1 Domain

The TSP-1 domain consists of a fragment, from Asn198 to Glu243, of CTGF. The TSP-1 domain was found to interact with integrins α_3_β_1_ and α_3_β_3_ to inhibit angiogenesis in endothelial cells [28,29]. In the previous study, the CTGF TSP-1 domain was found to interact with vascular endothelial growth factor 165 (VEGF165) to regulate the angiogenesis process [30,31]. In other cases, such as liver fibrosis, it was necessary for the TSP-1 domain to be combined with the IGFBP and VWC domains to promote the interaction between CTGF and slit guidance ligand 2 (SLIT2) in cultured hematopoietic stem cell (HSC) and fibrotic murine livers. As a consequence, SLIT2/roundabout (Robo) receptor signaling has the potential to be activated; this signaling is involved in various regulatory processes related to tissue damage, such as the recruitment of inflammatory cells, the promotion of angiogenesis, and the facilitation of fibrosis [32]. Estrogen receptors (ERs) are proteins found in cells that bind to estrogen, a vital hormone in the development and function of female reproductive organs. CTGF was also reported to interact with ERs through the TSP-1 domain, promoting the transcriptional regulation of estrogen signaling [33].

### 2.4. CT Domain

The amino acid sequence of the CT domain is the fragment of CTGF from Cys256 to Pro330. The CT domain is involved in CTGF regulation, including cell adhesion, migration, and proliferation [1]. The CT domain interacts with ECMs, growth factors, and cell surface receptors to regulate their downstream signaling pathways. For example, the CT domain can bind to integrins to promote cell adhesion and migration. An amino acid site in the CT domain, GVCTDGR, is a conserved binding site for integrin α_V_β_1_ and α_V_β_3_, which are two major ligand ECMs in the CT domain. The CT domain contains a fragment (GVCTDGR) which can be recognized by integrin α_V_β_1_. This interaction could be essential for stimulating the pro-fibrotic activity of CTGF in pancreatic stellate cells (PSC) [34]. α_V_β_3_ in hepatic stellate cells (HSCs) was reported to bind to the CT domain to promote the adhesion of HSCs. 

For other ECMs, such as fibronectin, the CT domain directly binds to the fibrin binding domain (NFBD) and gelatin–collagen binding domain (GCBD) [35]. Upon binding to fibronectin, CTGF can be displayed on this protein, which can also affect cell adhesion and migration [36]. Wnt inhibitory factor 1 (Wif-1) is a negative regulator in the Wnt signaling pathway. Wif-1 binds to CTGF at the CT domain to inhibit the activity of CTGF and to reduce the expression of aggrecan and collagen II in primary murine chondrocytes [37]. The Wnt co-receptor, low-density lipoprotein receptor-related protein 6 (LRP6), is also an essential regulating factor in the Wnt signaling pathway. The CT domain was found to interact with LRP6 to promote the inhibition of Wnt signaling. In addition, this domain was found to be required for the interaction of CTGF with Frizzled8 [38]. At the neuromuscular junction (NMJ), the CT domain can recognize the third beta-propeller domain of the lipoprotein-related receptor (LRP4), enhancing the interaction between LRP4 and the muscle-specific receptor tyrosine kinase (MuSK). As a result, MuSK phosphorylation and acetylcholine receptor (AChR) expression are promoted in the neuromuscular junction of the skeletal muscle [39,40].

**Figure 2:**
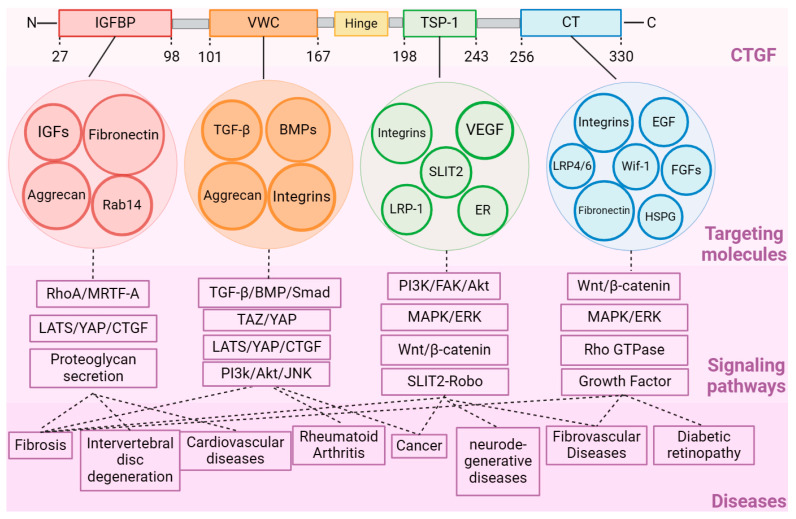
Diagram of the interaction network between CTGF’s domains and different molecules, signaling pathways, and diseases. Abbreviations: BMP, bone morphogenetic proteins; CT, cysteine knot-containing domain; EGF, epidermal growth factor; ER, estrogen receptors; FAK, focal adhesion kinase; FGFs, fibroblast growth factors; HSPG, heparan sulfate proteoglycan; IGF, insulin-like growth factor; IGFBP, insulin-like growth factor binding protein; LATS, long-acting thyroid stimulator; LRP, low-density lipoprotein receptor-related protein; MRTF-A, myocardin-related transcription factor A; RhoA, ras homolog family member A; SLIT2, slit guidance ligand 2; TGF-β, transforming growth factor-β; TSP-1, thrombospondin type-1 repeat; VWC, von Willebrand factor type C repeat; Wif, Wnt inhibitory factor; YAP, Yes-associated protein.

The interaction between the CTGF domains and different molecules is a complex network involving various diseases and physiological processes (Figure 2) (Table 1). The IGFBP and CT domains were found to jointly interact with the small leucine-rich proteoglycan protein Tsukushi to further regulate related physiological processes, such as early body formation, bone growth, wound healing, and retinal stem cell regulation [18]. Some studies have also discovered the binding of interesting molecules to CTGF, although the specific binding domain has not yet been investigated. For instance, the angiotensin-converting enzyme 2 (ACE2) receptor was reported to interact with CTGF in the viral infection process of severe acute respiratory syndrome (SARS)-CoV-2 [41]. In addition, increased mRNA levels of ACE2, CTGF, and fibronectin were also found and were regarded as drivers of lung fibrosis. SARS-CoV-2 binds with ACE2, promoting its interaction with CTGF and activating lung fibrosis processes [41]. In atherosclerotic lesions, the integrin αmβ2 was identified as an adhesion receptor for cysteine-rich 61 (Cyr61) and CTGF.

## 3. Roles of CTGF in Different Diseases

### 3.1. Fibrosis-Related Diseases in the Tissues

The aberrant expression and dysfunctional regulation of CTGF have been linked to several diseases, such as fibrosis, cancer, cardiovascular diseases, and diabetes [42,43,44,45]. Fibrosis is a pathological phenomenon that arises due to the excessive deposition of the extracellular matrix in different organs, leading to the formation of fibrous tissue and ultimately resulting in impaired function. It is characterized by a dysregulated tissue repair process induced by many types of tissue damage, especially in chronic inflammatory diseases. In the tissue repair process, local fibroblasts can be stimulated by damage-induced inflammatory monocytes and tissue-resident macrophages. The infiltrated macrophages promote the production of pro-inflammatory cytokines, such as IL-17, which was found to be a critical activator for the expression of TGF-β [46]. In turn, TGF-β promotes the expression of IL-17, IL-1, IL-6, and TNF, thus further stimulating the progression of inflammation and downstream fibrosis [47]. As a critical factor in TGF-β/Smad signaling, CTGF expression can be significantly up-regulated to stimulate the production and accumulation of the pro-fibrotic ECM proteins. When severe damage occurs, the accumulation of ECM proteins can be accomplished quickly, leading to the destruction of the tissue structure, organ dysfunction, and organ failure [48]. 

CTGF has been identified as a critical mediator in the development and progression of fibrosis, a condition characterized by excessive scarring and tissue damage [49,50]. The pathological process of fibrosis can be detected in various tissue diseases, including cardiac, liver, lung, kidney, muscle, and skin diseases. Investigating the basal expression level of CTGF in different tissues is crucial to understanding its role in tissue homeostasis and pathophysiology. Enhanced protein levels of CTGF have been observed in patients with fibrotic diseases such as pulmonary fibrosis, liver fibrosis, and kidney fibrosis [12,32,43,51]. Idiopathic pulmonary fibrosis (IPF) is a chronic progressive lung disease characterized by the formation of fibrotic scars in the lungs. In animal models, CTGF can induce severe fibrotic effects that cause significant pulmonary fibrosis under the administration of TGF-β [12]. The ECM expression level was significantly reduced CTGF-specific antibodies were administered in a pulmonary fibrosis animal model; consequently, the survival rate was improved [52]. In the healthy kidney, CTGF is produced at a low level in normal conditions. Its expression is up-regulated during the development of glomerulonephritis by the induction of inflammatory and fibrotic processes, indicating that CTGF is a potential target in the treatment of glomerulonephritis [51]. CTGF also plays essential roles in the development of renal fibrosis, such as in the stimulation of extracellular matrix deposition and the induction of mesangial cell cycle arrest, hypertrophy, and inflammation [53]. The inhibition of CTGF with an inhibitor and a neutralizing antibody could prevent the expansion of fibrotic ECMs and attenuate the progression of diabetic nephropathy in animal models. 

P53 is a factor found to cause elevated expression and accumulation in the hepatocytes of individuals with fibrotic liver diseases. In fibrotic human liver samples, an increased correlated gene expression between CTGF and p53 was observed in fibrotic livers. A high level of CTGF was mediated by p53 to repress the level of miR-17-92 and to induce liver fibrosis. In this fibrosis model, the p53/CTGF pathway could be a potent therapeutic target in the treatment of liver fibrosis [54]. In osteoarthritis, CTGF is regarded as a significant factor in synovial fibrosis. The overexpression of CTGF by the adenovirus vector can further exacerbate synovial fibrosis in the mouse knee synovium [55,56]. Duchenne muscular dystrophy (DMD) is a severe muscle-wasting disease characterized by progressive fibrosis in muscle cells, such as those of the skeletal muscle, soleus muscle, and diaphragm. Patients with DMD are more likely to be affected by muscle damage, which leads to progressive loss of muscle tissue and physiological function [57]. In the DMD and *mdx* mouse model (a dystrophin-null mouse model), CTGF was increased in the skeletal muscle fibers with elevated levels of ECM protein deposition, such as that of fibronectin, collagen, and alpha-smooth muscle actin (α-SMA). As with the CTGF hemizygous deletion mice, the *mdx* mice treated with CTGF-specific antibody exhibited improved muscle strength and less skeletal muscle damage compared to the *mdx* control mice [17,58], which implies that CTGF is a potent therapeutic target in the regulation of the progression of fibrosis in DMD.

### 3.2. Cancer Diseases

Cancer is a leading cause of death worldwide. More than 35 million new cancer cases are expected in 2050, representing a 77 percent increase compared to the 20 million estimated in 2022. CTGF is a promising cancer therapeutic target with great potential to improve the clinical outcomes for cancer patients. For most cases, CTGF has been shown to have pro-tumor effects. It is commonly reported that high levels of CTGF actively participate in the process of initiation, development, metastasis, proliferation, migration, invasion, drug resistance, and epithelial–mesenchymal transition (EMT) [2]. Triple-negative breast cancer (TNBC) is clinically characterized by its high aggressiveness and limited treatment options due to the existence of several subtypes, necessitating the identification of new therapeutic targets [59]. The level of CTGF is negatively correlated with the patient survival rate in this cancer. CTGF overexpression promotes cell proliferation in triple-negative breast cancer (TNBC) cells and triggers migration by activating integrin/ERK signaling. Similarly, the depletion of CTGF caused a depression in cell proliferation, lactate, and ATP production in a TNBC model [60,61]. CTGF knockdown caused by siRNA transfection significantly reduced the enrichment and expression of the gene sets, including ECM–receptor interaction, ECM organization, cell adhesion, focal adhesion, and chemotaxis in the MDA-MB 231 cells (a typical TNBC cell line), which suggested that CTGF could also be a potent prognostic marker in TNBC treatment [60]. 

Colorectal cancer is the third most common form of cancer, with a 5-year survival rate of about 14% [62]. Many cell samples of patients suffering from colorectal cancer exhibit epithelial–mesenchymal transition (EMT), which is a fundamental process in embryonic development and involves the transformation of polarized epithelial cells into active mesenchymal cells. Cancer cells express high levels of EMT-promoting factors, making EMT an essential process for invasiveness; EMT is associated with tumor progression, metastasis, and drug resistance [63,64]. As a downstream effector of TGF-β, CTGF could promote EMT in combination with TGF-β in a dose- and time-dependent manner. An attenuated level of CTGF was also found to partially reverse EMT progression in human peritoneal mesothelial cells [65]. The up-regulation of CTGF could promote the EMT process in colorectal cancer and even rescue the suppression effects on EMT and angiogenesis caused by the inhibitor miR-218 [66]. 

Globally, gastric cancer is the fifth most common form of cancer, and it is the third leading cause of cancer diseases. Patients with elevated CTGF expression exhibit significantly lower cumulative postoperative 5-year survival rates than patients with regular or reduced CTGF expression [67]. In the microenvironment of gastric carcinoma cells, abnormal matricellular protein secretion can occur in the tumor cells and in the surrounding stromal cells to promote carcinogenesis and regulate metastasis. As members of the matricellular protein family, CTGF and its inducible ECM proteins have essential roles in the carcinogenic effect of gastric cancer [68]. In gastric carcinoma cells, the overexpression of CTGF was observed, which promoted cell migration and metastasis in vivo [67]. Similarly, the knockdown of CTGF expression could inhibit the growth, migration, invasion, and peritoneal dissemination of gastric cancer in vivo [69]. CTGF enhanced cell resistance to fluorouracil (5-FU) in colon cancer, protecting tumor cells from apoptosis [70]. 

Bladder carcinoma is the ninth most common cancer and is the most epidemical malignant tumor in the urinary system [71]. It is characterized by high heterogeneity; thus, clinical treatment is challenging, and drug efficacy is limited. Based on this, a potent treatment strategy involves targeting the tumor microenvironment in bladder carcinoma. CTGF was found to be produced in the cancer-associated fibroblasts that consist of a tumor microenvironment component, which is related to tumor recurrence [72]. In a previous study, the components in the cancer-associated fibroblasts were primarily up-regulated in cancer. In particular, the expression level of CTGF and its up-regulating factor TGF-β1 in the cancer-associated fibroblasts was investigated and was found to be elevated in recurrent bladder carcinoma. Thus, targeting or inhibiting the CTGF protein may also have the potential to reduce bladder carcinoma recurrence [73]. 

### 3.3. Neurodegenerative Diseases

The progressive loss of neurons characterizes neurodegenerative diseases and is a growing cause of mortality, morbidity, and cognitive impairment, especially in older people. CTGF has emerged as a critical factor in the pathogenesis of neurodegenerative diseases [74,75]. In the central nervous system, CTGF primarily localizes and exerts immunogenicity in the astrocytes and a subpopulation of pyramidal neurons. In the central canal of the spinal cord and cerebral ventricle, CTGF also exhibits detectable immunoreactivity. Alzheimer’s disease (AD) is the most common neurodegenerative disorder and is characterized by the accumulation of amyloid-beta (Aβ) plaques and neurofibrillary tangles in the brain [76]. CTGF is highly expressed in the brains of individuals and mouse models with AD [77]. The expression of CTGF, which is elevated in the vicinity of amyloid-beta (Aβ) plaques and neurofibrillary tangles (NFTs), has been shown to exert an influence on γ-secretase activity, the pivotal molecular process involved in the production of Aβ [78]. CTGF may potentially facilitate an increase in the stability of amyloid-beta through a mechanism involving the activation of gamma-secretase and the reduction in the insulin-degrading enzyme (IDE), which depends on MAP kinase (MAPK)/phosphatidylinositol 3-kinase (PI3K)/protein kinase-B (AKT) [79]. The expression of CTGF in the brain could potentially serve as a novel therapeutic target for the clinical progression and neuropathology associated with Alzheimer’s disease.

Globally, Parkinson’s disease is the second most common serious neurodegenerative disease; it involves a loss of dopaminergic neurons and is highly pathogenic to millions of people aged 60 years and above. This disease is characterized by the progressive loss of dopaminergic neurons in the substantia nigra, leading to motor and non-motor symptoms [80]. Neuroinflammation can significantly contribute to progressive dopaminergic (DA) neurodegeneration in Parkinson’s disease. In this disease, the expression of CTGF was also found to be increased in an LPS-induced male Sprague Dawley rat model of the substantia nigra region, which indicated that CTGF may participate in neuroinflammation-induced DA neurotoxicity [81]. 

Amyotrophic lateral sclerosis (ALS) is a fatal neurodegenerative disease of the central nervous system that occurs with age and is highly recognized in patients of around 60 to 79 years old [82]. ALS is characterized by a combination of upper (UMN) and lower motor neuron (LMN) dysfunctions that affect the bulbar, cervical, thoracic, and lumbar segments [83]. According to a previous study, the expression of CTGF was increased in the amyotrophic lateral sclerosis human spinal cord, astrocytes, and neurons [84]. By inhibiting the activity of CTGF through the monoclonal neutralizing antibody FG-3019, muscle atrophy and fibrosis in skeletal muscle were reduced in hSOD1G93A mice [84].

### 3.4. Ophthalmic Diseases

Vision impairment and blindness caused by diabetic retinopathy, glaucoma, age-related macular degeneration, refractive errors, and cataracts can have severe consequences for the affected individual. In ophthalmic diseases, CTGF is highly expressed in patients with retinal diabetes, retinal vascular dysfunction, and inflammation [85,86]. Diabetic retinopathy is a significant complication that occurs in about 30–40% of diabetic patients. It is also a primary cause of blindness and visual impairment. In the early stages of diabetic retinopathy, CTGF is associated with basal lamina thickening and pericyte loss [87,88]. Basal lamina thickening could contribute to the progression of early diabetic retinopathy. The main components of retinal capillary basal lamina are collagen, laminin, and fibronectin. These components are also vital downstream ECM proteins that are regulated by CTGF. When an imbalance between the synthesis and degradation of the ECM components occurs, the retinal capillary basal lamina is likely to be impaired. In experimental models, reducing the level of ECM components in basal lamina prevented its thickening and further inhibited the process of diabetic retinopathy [89]. Pericyte loss is another crucial phenomenon related to diabetic retinopathy. The loss of pericytes can cause impairment of the capillary structure, blood flow, and blood–retinal barrier. In an experimental diabetes mouse model, the depletion of CTGF was found to attenuate pericyte loss, which served as further proof of the regulating function of CTGF in diabetic retinopathy [88]. In the late clinical stages, CTGF contributes to the progression of vascular fibrosis [90,91]. Vitreous hemorrhage caused by vascular fibrosis is one of the pathological processes in diabetic retinopathy. In addition, CTGF-induced fiber vascular membrane formation is an essential cause of the process of vascular fibrosis. In an experimental study, an elevated CTGF protein level was also investigated in the fiber vascular membrane [92]. Thus, by modulating vascular fibrosis, CTGF could stimulate the process of diabetic retinopathy. 

Age-related macular degeneration (AMD) is another leading cause of blindness in older people. Neovascular AMD (nAMD), namely wet AMD, is a major type of AMD and can be treated with an anti-VEGF drug in many patients. However, approximately 30% of nAMD individuals may sustain irreversible visual damage, subretinal fibrous scar formation, and subretinal hemorrhaging due to subretinal fibrosis. CTGF is involved in the proliferation, migration, matrix synthesis, and fibrosis of the RPE cell, which is an essential factor in the promotion of the process of subretinal fibrosis [93]. Thus, CTGF might be a potential target in the treatment of nAMD with subretinal fibrosis. In a previous study, an up-regulated level of CTGF was found in the retinal pigmented epithelial (RPE) cell, which could be a significant cause of the induction of AMD in patients [94]. Additionally, an effective drug candidate was reported to inhibit subretinal fibrosis by suppressing the expression of CTGF in an nAMD experimental model [95]. 

Corneal fibrosis is a process of excessive healing of the corneal stroma. Keratitis caused by infectious exposure or physical or chemical damage can lead to persistent fibrosis of the cornea, which leads to a loss of corneal transparency and blindness [96]. CTGF can improve the production of fibrosis markers, leading to retinal fibrotic lesions and mediating the degree of fibrosis of the corneal fibroblasts. In patients with severe corneal fibrosis, the expression of CTGF is promoted by TGF-β; thus, the level of fibronectin and collagens in the corneal fibroblasts increases [97].

Glaucoma is a condition causing irreversible vision loss and is characterized by a progressive loss of retinal ganglion cells. In severe cases, it can lead to irreversible blindness [98]. CTGF has also been associated with glaucoma characterized by optic neuropathy [99,100]. In patients with glaucoma, the basal levels of CTGF are higher than in healthy individuals [99]. In other cases, deposit formation, inflammation, and fibrosis are also related to severe blindness in ophthalmic diseases [101]. For instance, proliferative vitreoretinopathy (PVR) is a significant cause of contractile preretinal membrane (PRM) formation and exhibits the possibility of causing blindness. In PVR, CTGF was able to promote the progression of EMT and the production of ECM proteins by regulating PI3K/Akt signaling [102]. In this model, the synthesis of collagen was reduced when cells were treated with 8-Br-cAMP to inhibit the level of CTGF [102]. 

### 3.5. Cardiovascular Diseases

Cardiovascular disease (CVD) can seriously impact heart health and vascular health and causes a group of disorders of the heart and blood vessels; it is one of the leading causes of human death globally. In this field, CTGF is related to atherosclerosis, hypertension, heart failure, and cardiovascular disease development [103,104]. 

In the blood vessels, CTGF promotes the growth of smooth muscle cells and the deposition of ECM proteins, leading to vessel narrowing and impaired blood flow [105]. Kawasaki disease (KD) is an acute vasculitis disease that is the primary cause of pediatrically acquired heart disease globally. In this disease, a lower level of Krüppel-like factor 4 (KLF4) in vascular endothelial cells (ECs) can cause a reduction in the miR-483 level and elevate the synthesis of CTGF, thereby enhancing the process of endothelial-to-mesenchymal transition (EndMT) in patients with acute KD [106]. When treated with atorvastatin, the level of CTGF was reduced, which suggested that CTGF could be a target for KD therapy [106]. Myocardial infarction is a severe cardiovascular disease that causes damage to the myocardium when the blood supply to the coronary arteries is disturbed. CTGF mRNA levels significantly increased within 72 h of reperfusion in the infarcted murine myocardium, further stimulating collagen and fibronectin expression in this model [103].

Hypertension is an essential cause of the development of cardiovascular diseases. Gradually, vascular remodeling is being considered as a leading factor of hypertension that could be regulated by CTGF. In a previous study, the proliferation of vascular smooth muscle cells (VSMCs) was promoted by elevated expression levels of CTGF in the hypertension process [105]. Under other conditions, CTGF also affects the regulation of VSMC proliferation, causing hypoxia, high glucose pressure, and advanced glycation end-products [107,108,109]. Thus, CTGF could be a regulation switch to control the degree of vascular homeostasis, which suggests that CTGF is a potential target in the treatment of hypertensive vascular diseases. Atherosclerosis is related to vascular fibrosis and is one of the main causes of cardiovascular disease. The process of vascular fibrosis includes the production and accumulation of ECMs, contributing to the stability of the vascular structure and to scar formation [110]. In the vasculature, CTGF is up-regulated in advanced atherosclerosis in human patients, which implies that CTGF is a potential target in atherosclerosis [111]. 

## 4. CTGF Targeted and Inhibitory Drug Discovery

Targeting CTGF to affect and inhibit its associated pathways provides a promising approach for therapeutic intervention for various diseases. In previous studies, researchers have developed small molecules, biologics, and novel therapeutic modalities such as monoclonal antibodies, peptides, nucleotide-based inhibitors, compounds, adeno-associated viral (AAV)-based therapy, and gene editing to modulate the expression of CTGF and its downstream signaling pathway [43,112,113,114]. 

Small molecules are organic compounds that can be orally administered and possess the ability to penetrate cell membranes. These molecules have been developed as inhibitors to disrupt CTGF signaling pathways and impede their pathological activity. Curcumin, which is one of the small molecules prepared from natural sources, is a curcuminoid of turmeric. This molecule was found to significantly inhibit hepatic stellate cell-induced angiogenesis by reducing the expression of CTGF. It could potentially be used to block the interaction between tumors and their associated stromal cells [115]. Letrozole is a potent aromatase inhibitor that targets and inhibits the aromatase enzyme and may be available for the adjuvant treatment of breast cancer. In CTGF-mediated diseases, letrozole could relieve the process of liver fibrosis by inhibiting the YAP-CTGF pathway to reduce the level of CTGF and suppress the retinoic acid in the hepatocytes [116,117]. Sunitinib is a multikinase inhibitor that could be used as a first-line treatment for clinical cancers. In a cardiomyocyte model, sunitinib could induce MHGB1-dependent autophagy to promote the degradation of CTGF [118]. Although this study aimed to reveal the toxic effects of sunitinib, the revealed mechanism of CTGF degradation by this compound is worth considering as a reference in the development of CTGF-inhibitory candidates in the future.

As CTGF is an extracellular secreted protein in the circulatory system, high-molecular-weight antibodies can specifically target and inhibit the extracellular CTGF protein, which makes the CTGF-specific antibody (FG-3019, namely Pamrevlumab) an ideal drug candidate for the treatment of CTGF-related diseases. FG-3019 is a human-sourced recombinant monoclonal antibody targeting the VWC domain of CTGF at the amino acid sites between Cys142 and Gly157 [119]. This antibody is generated to neutralize CTGF activity and to create the disease symptoms in mouse models and clinical trials, including those of muscular dystrophy, liver fibrosis, diabetes, and idiopathic pulmonary fibrosis [2,43,84,120]. For tumor therapy, FG-3019 can promote an inhibition effect on the progression of pancreatic cancer, lymphoblastic leukemia, ovarian cancer, and melanoma in experimental models [121,122,123]. For the treatment of idiopathic pulmonary fibrosis and Duchenne muscular dystrophy, FG-3019 has obtained the orphan drug designation from the U.S. Food and Drug Administration [56]. In addition to FG-3019, a single-chain fragment variable (scFv) with a high affinity for human CTGF was obtained in a previous study using phage display technology [124]. This scFv also exhibited a high level of affinity for CTGF, with a dissociation constant of 0.782 nM. Via administration in a collagen-induced arthritis (CIA) mouse model, the scFv was found to relieve arthritis and reduce levels of pro-inflammatory cytokines [124]. Unlike the VWC binding domain of FG-3019, the scFv could target the TSP-1 domain, which suggests that these two types of antibodies may have distinct therapeutic effects in the different regulating processes of CTGF. For instance, the VWC domain could be associated with TGF-β related fibrosis signaling, while the TSP-1 domain could be related to cell adhesion and tissue repair. Different choices of antibodies may obtain distinct therapeutic effects.

Designed synthetic peptides are also a component of the CTGF-specific targeting strategy. The CTGF-specific peptide in the pancreatic ductal adenocarcinoma could change the tumor microenvironment and inhibit tumor growth in a dose-dependent manner [125]. To treat kidney fibrosis in chronic kidney disease, a novel CTGF–CT domain-specific peptide, named 810A, was developed to block the interaction between CTGF and its epidermal growth factor receptor, thereby inhibiting the STAT3 phosphorylation and cellular ECM protein synthesis to alleviate the process of kidney fibrosis [126]. As MMP cleavage sites exist at the linker between CTGF domains, CTGF is susceptible to MMP cleavage in the extracellular matrix. Therefore, the process of CTGF activity may not only be performed by the full-length CTGF protein, but may also include the effects of individual domains. The antibody FG-3019 could specifically bind to the VWC domain of the full-length CTGF protein and the individual VWC domain. In contrast to the FG-3019 antibody, peptide 810A could block the interaction of both full-length CTGF and the CT domain with EGFR. This CTGF–CT domain-specific peptide might be a more suitable candidate in the treatment of EGFR/CTGF-induced kidney fibrosis compared to FG-3019.

Nucleotide-based therapies, such as antisense oligonucleotides (ASOs), small interfering RNAs (siRNAs), and specific DNA/RNA aptamers, have emerged as promising approaches for the targeting of CTGF [127,128,129]. It is worth noting that nucleotide-based therapies have the potential to target intracellular CTGF, in contrast to high-molecular-weight candidates, such as antibodies. Because small-molecule nucleic acid drugs have the ability to enter cells, intracellular CTGF can be more efficiently targeted and inhibited by combining it with cell-penetrating functionalized nanoparticles or transfection reagents. RNA interference is characterized by the specificity and efficiency of gene inhibition through short double-stranded RNA (siRNA). In the disease process of spinal cord injury, the conditional depletion of CTGF can reduce the proliferation of glial scars. To penetrate the blood–spinal cord barrier, CTGF-targeting siRNA was constructed in combination with a mesenchymal stem cell-derived exosome for the treatment of spinal cord injuries. In this study, exosome-based siRNA was found to significantly inhibit CTGF expression, attenuating inflammation, neuron apoptosis, and glial scar formation [129]. Wound healing usually results in skin scarring, which affects the normal functional structures of the skin, such as the hair follicles, sweat glands, and nipples. The continuous inhibition of CTGF helps to control the excessive fibrotic response in skin wound healing. In one study, using an siRNA drug candidate in a rat burn model, CTGF was successfully inhibited, which in turn affected the expression of collagen I and α-SMA, causing scar reduction and promoting normal skin function [130]. In the field of the development of CTGF-targeting siRNA, OliX Pharmaceuticals has established a series of candidates, such as OLX101, OLX201, OLX301, and OLX701, which were used in the treatment of hypertrophic scars, idiopathic pulmonary fibrosis, macular degeneration, and liver fibrosis [131]. 

ASOs can be administered to interfere with RNA processing and to influence the protein expression process. The suppression of target protein by ASOs can be performed by blocking protein translation or affecting the splicing to introduce an out-of-frame depletion, thereby leading to the knockdown of the targeted molecule [132]. To treat fibrosis and scar formation, an ASO candidate, named EXC-001, was established; it was reported to alleviate collagen accumulation and scar formation by inhibiting CTGF expression [128]. Unlike the siRNA and ASO candidates that could only affect nucleic acid targets, nucleic acid aptamers, as a type of highly specific single-stranded DNA/RNA, targeted a variety of molecules of interest, including proteins, compounds, peptides, and nucleic acids [133]. In a previous study on the treatment of a collagen-induced arthritis mouse model, a highly specific (dissociation constant of 7.86 nM) aptamer candidate was obtained through a protein-based systematic evolution of ligands using an exponential enrichment (SELEX) aptamer screening process. This aptamer exhibited antiproliferative and antiangiogenic activities and had therapeutic effects on joint injury and the inflammatory response [112].

Gene therapies involve the introduction of the expression or the editing of vectors into cells to modulate gene expression. The suppression of the CTGF gene was found to be therapeutically effective in animal models using lentivirus vector and AAV-based CRISPR-Cas9 technology [113,134]. Endogenous host miRNAs that are capable of inhibiting CTGF were identified as potent candidates for CTGF-targeted drug discovery during the investigation of the CTGF mechanism. Manipulating the expression of miRNAs may provide a novel targeted therapeutic strategy. In a previous study, miR-26 was a reported candidate that exhibited the inhibition of proliferation and promoted the apoptosis of human Tenon capsule fibroblasts by suppressing the expression of CTGF [135]. MiR-113a was identified as a potent factor in the inhibition of the fibrotic activity of TGF-β1 and CTGF in myofibroblast differentiation and pulmonary fibrosis [136].

## 5. Non-Clinical and Clinical Trials of CTGF-Targeted and Inhibitory Therapy

The laboratory-developed drugs for targeted CTGF therapy demonstrate more significant differentiation in their efficacy in various disease models than in clinical trials, especially phase II and phase III clinical trials. From lab experiments to clinical trials, many specific candidates have been developed with various efficacies, including small-molecule compounds, antibodies, siRNA, and stem cells [43,137,138,139]. Continually focusing on the subsequent development of candidates in clinical trials helps to foster a better understanding of the potential challenges and issues that occur during the clinical and industrial translation phase (Table 2).

The CTGF-specific antibody FG-3019 has been used in many published studies and is currently undergoing clinical trials for the treatment of fibrosis-related diseases. This antibody has been shown to reduce the progression of idiopathic pulmonary fibrosis (IPF) and to improve lung function [43]. In the treatment of Duchenne muscular dystrophy (DMD), FG-3019 was found to reduce muscle dystrophy in the *mdx* mouse model [140]. In the amyotrophic lateral sclerosis (ALS) mouse model, FG-3019 reduced fibrosis in the skeletal muscle of hSOD1G93A mice and improved muscle and locomotor performance [84]. Regarding clinical trials, three studies have completed their phase II trials for the treatment of IPF (NCT01890265, NCT01262001) and COVID-19 pneumonia (NCT05262309). However, regarding phase III clinical trials, just one completed trial can be found for the treatment of COVID-19 pneumonia and three active trials can be observed for advanced pancreatic (NCT03941093), metastatic pancreatic cancer (NCT04229004), and ambulatory DMD (NCT04632940). It is disappointing that the phase III ZEPHYRUS-1 trial of FG-3019 in patients with IPF failed to meet its primary endpoint (NCT03955146); it also failed to achieve its primary goal in a trial for advanced DMD (NCT04371666), and even the Phase III ZEPHYRUS-2 trial for IPF was terminated (NCT04419558). Nevertheless, it is worth paying attention to the data from two pancreatic cancer trials using FG-3019, which are expected to occur in 2024. 

**Figure 3:**
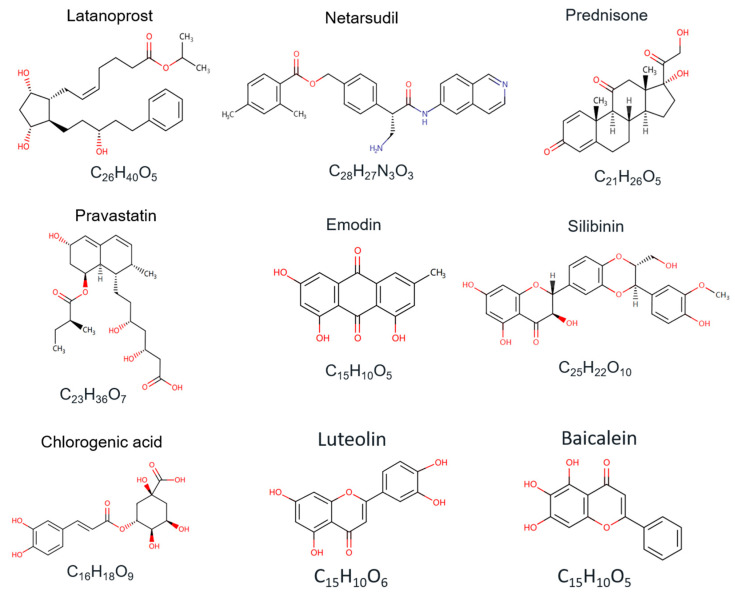
Structures of marketed compounds for CTGF-targeted and -inhibitory therapy. The chemical structures were obtained from the Drugbank online database (https://go.drugbank.com/, accessed on 7 April 2024).

Small-molecule compounds are the main candidates for CTGF-targeted therapy in drug development. The structures of several chemical and natural compounds are also listed for a better understanding (Figure 3). The marketed drug candidates, such as Latanoprost, Netarsudil mesylate, and Prednisone, have been approved for the treatment of CTGF-related glaucoma, hypertensive eye disease, corneal edema, Duchenne muscular dystrophy, prostate cancer, and autoimmune hepatitis [141,142]. Intracellularly, siRNA involves the introduction of synthetic or chemically modified double-stranded RNA molecules to target and inhibit specific gene expression. Treatment with CTGF-targeted siRNA has been found to treat the indications of pulmonary fibrosis and skin scarring in the clinical stages [139]. Stem cell therapy has been proven to be a promising strategy in the treatment of CTGF-related diseases by differentiating cells that can repair damaged tissue and inhibit the progression of fibrosis [143]. Stem cells release growth factors and cytokines when introduced into the affected area, stimulating tissue regeneration and suppressing inflammation to reduce fibrosis and improve organ function [144,145,146]. 

In addition to chemical compounds, naturally derived compounds are important sources of novel drug candidates. Natural-compound drugs are produced by extracting the endogenous active substances from a living organism in nature [147,148]. Because naturally extracted compounds demonstrate low side effects, these drugs have unique advantages in clinical use [149]. Plant extracts are the most common natural-product drugs and are effective candidates for the treatment of diseases such as CTGF-related fibrosis and heart failure. Chebulagic acid and chebulinic acid are alcoholic extracts of Triphala, which is a formulation of Ayurvedic herbal medicine. In the treatment of diabetic retinopathy, chebulagic acid and chebulinic acid could inhibit the expression level of fibrosis-related proteins (CTGF and ECM protein) by suppressing ERK/TGF-β signaling. Although CTGF is not their direct target, they also have a significant inhibitory effect on CTGF expression levels [150]. Emodin is a vital component extracted from Chinese herbal rhubarb, which contributes to a reduction in the progression of renal failure. By inhibiting the deposition and accumulation of Smad3 and CTGF, emodin has shown a therapeutic effect on renal tubulointerstitial fibrosis in obstructed kidneys [151]. Silymarin and caffeine are essential natural compounds for anti-inflammatory and antioxidant treatment in disease progression. Silibinin is extracted from Silybum and is a primary active compound of silymarin. The combined administration of silymarin and caffeine could prevent liver fibrosis by downregulating LPAR1, TGF-β1, and CTGF [152]. Chlorogenic acid is clinically used in the treatment of advanced cancer and impaired glucose tolerance. This compound can be extracted from plants such as bamboo, the shoots of heather, and hibiscus sabdariffa. By promoting the regulation effect of miR-21 on the TGF-β1/Smad7 signaling pathway, chlorogenic acid can inhibit the level of CTGF and fibrosis markers in the treatment of liver fibrosis [153]. 

**Table 2:** CTGF-targeting and CTGF-inhibiting candidates in clinical trials and marketing.

Candidates or Trial Titles	Targets	Indications	Status	Types	Ref.	ClinicalTrials ID.
Latanoprost	CTGF, PTGFR	Glaucoma, Hypertensive eye disease	Marketing	Chemical compound	[137]	NCT05283395
Netarsudil	CTGF, TGFBR2	Glaucoma, Hypertensive eye disease, Corneal edema	Marketing	Chemical compound	[138]	NCT03808688
Prednisone	GCR	Duchenne muscular dystrophy, Prostate cancer, Autoimmune hepatitis	Marketing	Chemical compound	[142]	NCT03439670
FG-3019	CTGF	Duchenne muscular dystrophy, Pulmonary fibrosis	Clinical Phase III	Monoclonal antibody	[58]	NCT04632940 NCT04419558 NCT04371666
OLX-10010	CTGF	Pulmonary fibrosis, Proliferative skin scarring	Clinical Phase II	siRNA	[139]	NCT04012099 NCT04877756
RXI-109	CTGF	Skin scarring, Age-related macular degeneration, Subfoveal choroidal Neovascularization, Subretinal scarring	Clinical Phase II	siRNA	[154]	NCT02079168 NCT02030275 NCT02599064
Pravastatin	TGF-β1, CTGF	Delayed cutaneous, Subcutaneous Radio-induced Fibrosis	Clinical Phase II	Chemical compound	[155]	NCT01268202
PRS-220	CTGF	Pulmonary fibrosis	Clinical Phase I	Chemical compound	[156]	NCT05473533

Abbreviations: PTGFR, prostaglandin F2alpha receptor; GCR, glucocorticoid receptor.

## 6. Conclusions and Perspectives

CTGF is an essential regulator in the pathogenic process of different diseases, making it a potential drug target candidate. This review summarizes the interactions between the CTGF domains and host factors and concludes with a summary of the regulation mechanisms in various disease models. The established CTGF-specific drugs in CTGF-related disease and drug discovery are also reviewed. 

The marketed drug candidates for CTGF-targeted therapy mainly include chemical compound hormones, such as hormone receptor agonists or inhibitors. Clinical treatments with hormone-related drugs are always challenged by various adverse effects that limit their clinical application. For the treatment of Duchenne muscular dystrophy, it is necessary for patients to use drugs, such as prednisone, for a long time. As a type of corticosteroid, unregulated doses or prolonged administration of prednisone can induce certain adverse effects, such as neuropathy, osteoporosis, peptic ulcers, and adrenal insufficiency. Regarding the other two marketed drug candidates for CTGF-targeted therapy, overdosing with latanoprost is not likely to induce extremely severe outcomes, but it can lead to conjunctival or episcleral hyperemia [157]. As with latanoprost, elevated lacrimation, erythema of the eyelid, and decreased visual acuity can occur in patients who receive netarsudil once per day. Thus, even though the most commonly used treatments in the clinic are still hormonal drugs, the development of other types of drug candidates with low adverse effects is still urgently required.

In clinical trials, although it is disappointing that the VWC domain-specific antibody (FG-3019) failed in the phase III clinical trial of DMD (https://firstwordpharma.com/story/5748991, accessed on 7 April 2024) and IPF (https://firstwordpharma.com/story/5754712, accessed on 7 April 2024), a large number of promising treatments have been reported in both non-clinical and early clinical laboratory studies. Furthermore, the knockdown of the CTGF gene or treatment with therapeutic hormone drugs has entered clinical trials and has even been used on the market, illustrating the potential of CTGF as a therapeutic target. As for the FG-3019 antibody, there are still exciting and ongoing clinical trials in the treatment of coronavirus pneumonia and pancreatic cancer.

Although large-molecule drugs have shown some efficacy in the targeting of extracellular CTGF [43,58], there may have been differences in the regulatory mechanisms of CTGF between the intracellular and extracellular environments. Although non-clinical and early clinical studies have shown the efficacy of the drugs targeting CTGF, the drug screening process and mechanism of action of CTGF may vary in different disease models. Understanding CTGF signaling in different disease conditions is necessary in the development of a particular CTGF-specific medicine to deal with specific disease conditions. For example, fibronectin is a critical CTGF-induced disease marker. In an ALS mouse model (hSOD1G93A), a high protein level of CTGF did not elevate the protein level of fibronectin, which indicated that CTGF might not participate in the regulation of fibronectin, although the diaphragm stress was promoted and the fibronectin protein level was inhibited by treatment with the CTGF-specific antibody (FG-3019) in this model [84]. This implies that CTGF might still be an unknown regulating mechanism in relation to diaphragm stress in ALS. However, in muscle fibrosis, the high level of CTGF was found to be associated with a high level of fibronectin. In a denervation-induced skeletal muscle fibrosis model, the antibody FG-3019 significantly inhibited the fibronectin protein level without elevating the muscle-specific force [158]. Thus, simply treating with FG-3019 may not have the ideal effect if the intention is to treat the disease by reducing FN. Therefore, it is necessary to consider the regulatory mechanisms of CTGF in different diseases comprehensively while developing CTGF-targeted drug candidates. 

The targeting of intracellular CTGF remains a field of research worth exploring. However, it is challenging for large molecules such as antibodies to penetrate the cell membrane and target intracellular CTGF, which might be one of the main reasons for FG-3019’s failure in DMD and IPF. While CRISPR or siRNA can inhibit the expression of intracellular CTGF genes, they do not directly target intracellular CTGF protein. Small-molecule drugs based on nucleic acids can be viable candidates for the targeting of intracellular CTGF. Functionalized and modified nucleic acid aptamers can penetrate the cell membrane via receptor-mediated endocytosis or micropinocytosis, making them more likely to target intracellular CTGF [159]. Nucleic acid aptamer modification techniques have fostered the ability of these drugs to exert some effects on targeted molecules in different cellular physiological processes. For example, aptamers are available candidates for the design and construction of targeted protein degradation (TPD) technology in cells. Through modification with proteolysis targeting chimeras (PROTACs), the aptamer–PROTAC complex can specifically bind to intracellular proteins and induce protein degradation through the ubiquitination system [160]. Thus, in the future development and marketing of clinical CTGF inhibitors, the possibilities of nucleic acid drugs, especially aptamer candidates, could be further studied, which would contribute to the related disease therapy.

The regulatory mechanism differences in the disease models need to be taken into account in the development of CTGF-targeted drugs. Continued research into CTGF inhibitory drugs may expand the choice of medications used in the treatment of fibrotic diseases such as hepatic fibrosis, pulmonary fibrosis, and cardiovascular fibrosis [12,32,161]. In addition, CTGF inhibitors may have critical paired therapeutic applications in cancer treatment, as they could be important mediators of angiogenesis and tumor growth [2,162]. The combination of CTGF inhibitors with other therapies could be developed in future studies to improve efficacy and to expand the scope of CTGF-targeted therapies. 

## Figures and Tables

**Table 1:** Summary of interactions between CTGF domains and different molecules.

Domains of CTGF	Targeting Molecules	Related Downstream Pathways of Targeted Molecules	Related Diseases Regulated by the Signaling Pathways
IGFBP	IGFs Fibronectin Aggrecan Rab14	RhoA/MRTF-A LATS/YAP/CTGF Proteoglycan secretion	Tissue fibrosis Intervertebral disc degeneration
VWC	TGF-β BMPs Integrins Aggrecan	TGF-β/BMP/Smad TAZ/YAP LATS/YAP/CTGF PI3k/Akt/JNK	Tissue fibrosis Rheumatoid Arthritis Cancer
TSP-1	Integrin α_3_β_1_ Integrin α_3_β_3_ VEGF LRP-1 SLIT2 ER	PI3K/FAK/Akt MAPK/ERK Wnt/β-catenin SLIT2-Robo	Fibrosis Breast Cancer Alzheimer’s Disease Fibrovascular Diseases
CT	Wif-1 EGF FGFs Integrin α_5_β_1_ Integrin α_5_β_3_ LRP-4/6 Fibronectin HSPG	Wnt/β-catenin MAPK/ERK Rho GTPase Signaling Growth Factor Signaling	Fibrosis Abnormal blood vessel growth Diabetic retinopathy

Abbreviations: EGF, epidermal growth factor; FAK, focal adhesion kinase; FGFs, fibroblast growth factors; HSPG, heparan sulfate proteoglycan; LATS, long-acting thyroid stimulator; MRTF-A, myocardin-related transcription factor A; RhoA, Ras homo-log family member A; YAP, Yes-associated protein.

## Abbreviations

ACE2, angiotensin-converting enzyme 2; AChR, acetylcholine receptor; AD, alzheimer’s disease; Akt, protein kinase B; ALS, amyotrophic lateral sclerosis; AMD, age-related macular degeneration; ASOs, antisense oligonucleotides; Aβ, amyloid-beta; BMP, bone morphogenetic proteins; CCN, communication network factor; CT, cysteine knot-containing domain; CTGF, connective tissue growth factor; CVD, cardiovascular disease; Cyr61, Cysteine-rich 61; DMD, Duchenne muscular dystrophy; ECM, extracellular matrix; ECs, vascular endothelial cells; EMT, epithelial–mesenchymal transition; ERK, extracellular signal-regulated kinase; ER, estrogen receptors; FN, fibronectin; GCBD, gelatin-collagen binding domain; HSC, hematopoietic stem cell; IDE, insulin-degrading enzyme; IGF, insulin-like growth factor; IGFBP, insulin-like growth factor binding protein; IPF, idiopathic pulmonary fibrosis; JNK, c-Jun N-terminal kinase; KD, Kawasaki disease; KLF4, Krüppel-like factor 4; LMN, lower motor neuron; LRP6, low-density lipoprotein receptor-related protein 6; MAPK, manner dependent on MAP kinase; MMP, matrix metalloproteinase; MuSK, muscle-specific receptor tyrosine kinase; NFBD, fibrin binding domain; NFTs, neurofibrillary tangles; NMJ, neuromuscular junction; PI3K, phosphatidylinositol-3 kinase; PRM, preretinal membrane; PSC, pancreatic stellate cells; PVR, proliferative vitreoretinopathy; RPE, retinal pigmented epithelial; SLIT2, slit guidance ligand 2; TGF-β, transforming growth factor-β; TNBC, triple-negative breast cancer; TSP-1, thrombospondin type-1 repeat; TβRII, transforming growth factor-β receptor II; UMN, upper motor neuron; VEGF, vascular endothelial growth factor; VWC, von Willebrand factor type C repeat; Wif, Wnt inhibitory factor.
